# Dietary ethanol ingestion by free-ranging spider monkeys *(Ateles geoffroyi*)

**DOI:** 10.1098/rsos.211729

**Published:** 2022-03-16

**Authors:** Christina J. Campbell, Aleksey Maro, Victoria Weaver, Robert Dudley

**Affiliations:** ^1^ Anthropology, California State University, Northridge, CA, USA; ^2^ Integrative Biology, University of California, Berkeley, CA, USA

**Keywords:** ethanol, spider monkey, drunken monkey hypothesis, primate frugivory

## Abstract

Ethanol within ripe and over-ripe fruit is produced naturally through the metabolic activity of fermentative yeasts. As a consequence, frugivorous animals may chronically consume ethanol as part of their routine diet, although direct measurements of such exposure are lacking. Here, we present data on ethanol concentrations within fruits of *Spondias mombin* (Anacardiaceae) that are eaten by black-handed spider monkeys (*Ateles geoffroyi*) on Barro Colorado Island, Panama. Of collected fruits that were partially consumed and then dropped by foraging monkeys, pulp-ethanol content was typically in the range of 1–2%; the percentage of pulp for consumed fruits was not significantly correlated with the ethanol concentration of the pulp remaining within each fruit. Urine samples from foraging spider monkeys were also evaluated for the ethanol metabolites ethyl glucuronide and ethyl sulfate; five of six samples tested positive for both compounds. In aggregate, these data indicate natural exposure to fruit-associated ethanol in a wild primate species.

## 1. Introduction

The ‘drunken monkey’ hypothesis posits that the proclivity of humans to consume alcohol stems from a deep-rooted affinity of frugivorous primates for naturally occurring ethanol within ripe fruit [1,2]. Ethanol is a by-product of fermentative yeasts within fruit pulp that metabolize sugar in the ripening process and that may serve as a long-distance olfactory cue to animal frugivores, including many primates. Human ancestors may also have preferentially selected ethanol-laden fruit for consumption (via short-range olfaction and also via gustation), given that its presence necessarily indicates co-occurrence of simple sugar precursors [3,4]. Psychoactive and hedonic effects of ethanol may similarly result in increased consumption rates and caloric gain. Contemporary patterns of alcohol consumption, in turn, may derive from these ancestral associations between ethanol and nutritional reward. Excessive consumption of alcohol, as with diabetes and obesity, can then be viewed conceptually as a disease of nutritional excess (see [2]).

Dietary ethanol is not avoided by wild mammals, at least not in low and naturally occurring concentrations. Wild slow lorises (*Nycticebus coucang*) and pen-tailed tree shrews (*Ptilocercus lowii*) chronically consume ethanol in the form of fermented nectar of the bertam palm (*Eugeissona tristis*), and occasionally do so at rates that would intoxicate humans ([5]; see also [6]). Feral vervet monkeys (*Chlorocebus aethiops*) introduced to Caribbean islands consume human-sourced alcoholic beverages [7]. Similarly, wild chimpanzees (*Pan troglodytes*) consume anthropogenically manipulated and fermented sap of the Raffia palm (*Raphia hookeri*), with ethanol concentrations as high as 6.9% [8]. Field trials with yeast-inoculated and ethanol-containing fruits also indicate that low concentrations correlate with enhanced dispersal by a variety of vertebrate frugivores [9], thereby falsifying the suggestion of Janzen [10] that ethanol at these levels is aversive (see also [11]). For frugivorous primates, however, there are at present no data available that directly demonstrate the consumption of fruits containing physiologically relevant ethanol levels.

Spider monkeys mostly consume ripe fruit [12] and as such are promising candidates for studies of natural ethanol exposure. For example, black-handed spider monkeys on Barro Colorado Island (BCI) often eat fruits of the palm *Astrocaryum standleyanum* [13], ripe fruits of which contain on average 0.5% ethanol in the pulp, and with over-ripe fruits containing about 4.5% [4]. Spider monkeys also possess excellent scent sensitivity to various alcohols, including ethanol, which may enhance foraging abilities [14]. Here, we examine the extent of ethanol in the diet of spider monkeys on BCI, and specifically of a major dietary item, ripe fruits of *Spondias mombin* (Anacardiaceae). On BCI, these fruits make up more than 5% of their total annual diet [13], and it is also important at other study sites throughout the range of *Ateles* spp. (e.g. [15–17]). The fruits have a high sugar content, with total non-structural carbohydrates equal to 40% of pulp dry weight [18], and are therefore likely candidates for alcoholic fermentation. These fruits are also used by indigenous human populations throughout Central and South America to make *chicha*, a fermented alcoholic beverage. We address the following questions: (i) Do *S. mombin* fruits consumed by spider monkeys contain measurable levels of ethanol?; (ii) Is there preferential consumption of those fruits with higher ethanol content? and (iii) Are secondary metabolites of ethanol present in the urine of free-ranging spider monkeys?

## 2. Material and methods

Fieldwork was conducted from June to September 2013, a period coinciding with the fruiting of *Spondias mombin*. Monkeys were located and then followed between the hours of 6.00 and 18.00. We opportunistically collected both non-consumed fruits (but observed to be rejected) and partially consumed fruits that fell to the ground during feeding bouts by monkeys in the canopy. Collected fruits were placed individually in plastic bags, stored on ice packs and were taken to the BCI laboratory within 6 h for analysis of ethanol and sugar content. Additional ripe fruits were also obtained from the ground beneath *S. mombin* trees with no spider monkeys present; unripe or over-ripe fruits (as characterized anthropogenically via colour and smell) were excluded. These ripe fruits were pooled for transport to the laboratory and were analysed in batches of seven fruits at a time.

For chemical analysis, we first removed the large seed from each fruit and then scraped all pulp from the seed and from the exocarp into a plastic weighing boat. The pulp was macerated, and sugar (i.e. total saccharide) content of the ground pulp was determined using a Westover RHB-32 Brix refractometer. Ethanol concentration and saccharide content of the pulp mixture were then determined via near infrared (NIR) spectrometry using the InfraSpec VFA-IR Spectrometer (Wilks Enterprise), which collects absorbance data at 0.00448 µm increments over the range of the 5.65–11.38 µm. A total of 232 individual calibration spectra was created to train the regression, with solute combinations (w/w) ranging from 0 to 20% saccharides of three types (i.e. glucose, fructose and sucrose), and 0 to 6.4% ethanol (see NIR calibration table in the electronic supplementary material). NIR spectra were analysed via a partial least-squares (pls) regression in R (v. 3.5.2), using the package ‘pls’. Rather than choosing the ‘best’ model using a single spectral range with a single specified number of principal components, we derived an average estimate (using a custom R script) for those pls models that met each of the following criteria: (i), fewer than 5% of all estimates yielded a negative ethanol concentration; (ii) the root mean squared error of prediction (RMSEP) for the bias-corrected cross-validation estimate (i.e. the adjusted coefficient of variation (adjCV) was always less than 0.6, and (iii) values of the RMSEP using the trained estimate from calibration data estimate were always less than 0.6. The threshold value of 0.6 for the RMSEP was based on a criteria-free initial analysis of a subset of all data, which yielded a minimum value of 0.56 for the adjusted coefficient of variation in RMSEP.

Additionally, we narrowed the spectral range of analysis to include only NIR wavelengths from 8.8 to 10.6 µm, which corresponds to minimal overlap between saccharide and ethanol absorbances and registers small differences in ethanol. For example, the analysis script began at a range of 8.8–9.7 µm and recorded results of any particular model (and a potentially variable number of principal components) that met the aforementioned inclusion criteria. The script then progressively shifted the spectral analysis window forward by one data point (i.e. by 0.00448 µm), until the upper bound of the range reached 10.6 µm. This process was then repeated using an initial window range of 8.8–9.70448 µm, yielding a total of 4104 spectral ranges each with at least one qualified model, from a possible 20 100 candidate ranges (i.e. approximately 20%). We then allowed the maximum number of possible principal components to be considered for each spectral range, resulting in a total of 114 539 models from a pool of greater than 4 million. We then aggregated the results in two different ways to estimate the ethanol concentration of any given sample. Method 1 first averaged all qualifying models for a given spectral range and then averaged the results for all spectral ranges; method 2 simply calculated the average of all the models, thus giving more weight to spectral ranges with a higher quantity of qualifying models. Both methods provided an estimate for the ethanol concentration of each sample as averaged over many regressions (see Results). The RMSEP adjCV for all models averaged 0.58 and ranged from 0.56 to 0.60% (i.e. less than half of the mean value for the overall mean for all samples). Both methods returned a negative estimate for only two (with a mean value of −0.07%) of all 110 evaluated fruit spectra.

Finally, we opportunistically collected urine samples from individual spider monkeys (located in the canopy) by holding beneath them a large plastic funnel attached to a 50 ml test tube. Urine samples were frozen until transported. Samples were shipped at room temperature from Panama to the United States (Panama export permit XEX/A-42–18; CDC import permit 2018-06-092) and were subsequently tested for ethyl glucuronide (EtG) and ethyl sulfate (EtS) at Southwest Labs (Albuquerque, NM, USA), using high-performance liquid chromatography/triple quadrupole mass spectrometry (MS/MS), as validated for human urine.

## 3. Results

Fruits of *S. mombin* that were partially consumed by spider monkeys contained measurable ethanol within the remaining pulp (i.e. from 1 to 2%; table 1). Similarly, the ethanol content of pulp obtained from fully intact and ripe fruits (but excluding unripe and over-ripe fruits) collected on the forest floor in the absence of foraging spider monkeys was near 2% (table 1).

**Table 1.** Mean (±s.d.) values for sugar and ethanol concentrations within pulp of inspected and either rejected (0% consumed) or partially consumed fruit (25%, 50% and 75% consumed), and for fallen ripe fruits with no spider monkeys present. For the last category, Brix data were available for only four of the six fruits analysed for ethanol content.

extent of consumed pulp	Brix (°Bx)	ethanol (%; method 1)	ethanol (%; method 2)
0% (*N* = 28)	12.81 (1.56)	1.55 (0.62)	1.67 (0.64)
25% (*N* = 68)	11.82 (1.31)	1.19 (0.56)	1.3 (0.63)
50% (*N* = 11)	12.79 (2.30)	1.28 (0.49)	1.39 (0.55)
75% (*N* = 2)	12.75 (1.77)	1.4 (0.34)	1.54 (0.42)
(fallen ripe fruits)	13.1 (1.44)	1.84 (0.35)	1.91 (0.36)

Of all rejected and partially consumed fruits (i.e. those with 25–75% fraction consumed), ethanol content estimated using method 1 was significantly different among the four consumption categories (ANOVA; d.f. = 3,102, *F* = 2.89, *p* < 0.04), with a significant interaction effect between % consumed and Brix value (d.f. = 3,102; *F* = 3.26, *p* < 0.03). *Post hoc* analysis showed ethanol levels (as calculated by method 1) to be significantly different only between the categories of 0% and 25% (Fisher protected least significant difference (PSLD), *p* < 0.003), with the former category higher on average by 0.32% ethanol (table 1). Similarly, for ethanol content estimated using method 2, the four consumption categories varied significantly (ANOVA; d.f. = 3,102, *F* = 2.81, *p* < 0.05), with a significant interaction effect between % consumed and Brix value (d.f. = 3,102; *F* = 3.1, *p* < 0.045). Of categorical comparisons, ethanol levels for rejected fruits were similarly higher than those of 25% consumed pulp (Fisher PSLD, *p* < 0.004), by about 0.30% ethanol (table 1). For neither method of ethanol estimation did the percentage of consumed fruit vary significantly with Brix value (*p* > 0.8 in both cases). Pooling both rejected (i.e. 0% consumed) and all partially consumed fruit samples, pulp-ethanol content and sugar concentration were weakly but positively correlated (method 1: *% ethanol* = −0.178 + 0.118 *Brix*, *R*^2^ = 0.101, *p* < 0.001; method 2: *% ethanol* = −0.095 + 0.121 *Brix*, *R*^2^ = 0.094, *p* < 0.002).

Of the six separate urine samples tested for ethanol metabolites, five tested positive for both EtG (with an average value of 458.02 ng ml^−1^) and for EtS (with an average value of 39.45 ng ml^−1^) (table 2). Three of the five positive EtG readings exceeded the cut-off (500 ng ml^−1^) set by Southwest Labs for clinical relevance in humans.

**Table 2.** Urine concentrations of EtG and EtS for six independent samples, as determined using liquid/chromatography/mass spectrometry.

sample ID (date)	EtG (ng ml^−1^)	EtS (ng ml^−1^)
110093 (2 July 2013)	592.68	47.12
110094 (2 July 2013)	0	0
110096 (24 June 2013)	752.95	60.70
110097 (16 June 2013)	301.20	29.59
110099 (14 August 2013)	317.0	37.73
110100 (13 July 2013)	784.34	61.56

## 4. Discussion

This study shows that spider monkeys on BCI ingest naturally occurring low-level ethanol as a constituent of one of their wild food items and is the first such demonstration for a frugivorous primate. Fruits with no evidence of consumption were probably smell-rejected and then dropped to the ground, but the possibility of accidental dislodgement during foraging cannot be excluded. Other cues such as texture, as assessed via palpation, and visual appearance may also have influenced rejection (see [19,20]). One limitation of this study is that fully consumed fruits cannot be analysed post-ingestion. However, the categories used here (table 1) nonetheless suggest no effect of ethanol content on pulp consumption once ingestion had commenced. Sugars and ethanol are comingled in ripe and fermenting fruit, and the significant interaction effects between the extent of pulp consumed and sugar level may simply indicate that spider monkeys prefer sweeter fruits with no direct assessment of ethanol content. Choice tests with captive spider monkeys [14] have demonstrated the ability to sense low-level ethanol, and to prefer ethanol-enriched sucrose solutions and ethanol-spiked pureed fruit, although strongly concentrated sucrose can override such preferences. The presence of ethanol at the naturally occurring pulp concentrations characterized here does not appear to be aversive to spider monkeys.

The presence of two ethanol-specific metabolites in urine samples of spider monkeys also is consistent with dietary exposure to this molecule. In humans, ethanol consumption typically yields elevated EtG and EtS levels for a number of days [21,22]. And for any given rate of consumption, urinary concentrations of both metabolites are strongly dependent on individual identity [23]. No relevant data exist for non-human primates that would enable the correlation of the EtG and EtS concentrations reported here to rates of ethanol ingestion, or to any behavioural or physiological consequences of such ingestion.

The sensory mechanisms by which spider monkeys, and frugivores more generally, localize ripe fruit are not well characterized with respect to their relative importance. Olfactory cues have been implicated in primates generally [24], and specifically in spider monkeys [20]. Given that ethanol is present in *S. mombin* fruits and presumably in other fruits of other tropical taxa, this volatile molecule may be important for foraging and localization at long distances, and also at short range (i.e. during selection of individual fruits for consumption). Ethanol may also act as a feeding stimulant, as it does in modern humans [25]. The demonstration here that ethanol is found within a major food item of a free-ranging primate raises the possibility of a number of behavioural consequences relevant to all frugivores, and as such merits further investigation. In particular, broadening the sample range to include a range of fruit ripening stages, interspecific comparisons of fruit-ethanol concentrations, and of associated ethanol consumption by a range of frugivorous primate taxa, would enhance our understanding of the role of this molecule in primate foraging biology. Given that positive selection on those genes encoding for ethanol catabolism has been substantial among fruit- and nectar-consuming mammalian species more generally [26], the natural consumption of fermented carbohydrates is likely to be more widespread than is currently realized.

## Data Availability

Code to analyze raw data in R, and accompanying data files, are available at Dryad Digital Repository, https://doi.org/10.6078/D1M420. Tables of calibration attributes and fruit attributes are available in electronic supplementary material [27].

## References

[1] Dudley R. 2000 Evolutionary origins of human alcoholism in primate frugivory. Q. Rev. Biol. **75****,** 3-14. (10.1086/393255) 10721531

[2] Dudley R. 2014 The drunken monkey: why we drink and abuse alcohol. Berkeley, CA: University of California Press.

[3] Dominy NJ. 2004 Fruits, fingers and fermentation: the sensory cues available to foraging primates. Integr. Comp. Biol. **44**, 295-303. (10.1093/icb/44.4.295) 21676713

[4] Dudley R. 2004 Ethanol, fruit ripening, and the historical origins of human alcoholism in primate frugivory. Integr. Comp. Biol. **44**, 315-323. (10.1093/icb/44.4.315) 21676715

[5] Wiens F, Zitzmann A, Lachance MA, Yegles M, Pragst F, Wurst FM, von Holst D, Guan SL, Spanagel R. 2008 Chronic intake of fermented floral nectar by wild treeshrews. Proc. Natl Acad. Sci. USA **105**, 10 426-10 431. (10.1073/pnas.0801628105) PMC2492458 18663222

[6] Gochman SR, Brown MB, Dominy NJ. 2016 Alcohol discrimination and preferences in two species of nectar-feeding primate. R. Soc. Open Sci. **3**, 160217. (10.1098/rsos.160217) 27493777 PMC4968469

[7] Palmour RM, Mulligan J, Howbert JJ, Ervin F. 1997 Of monkeys and men: vervets and the genetics of human-like behaviors*.* Am. J. Hum. Genet. **61**, 481-488. (10.1086/515526) 9326311 PMC1715973

[8] Hockings KJ, Bryson-Morrison N, Carvalho S, Fujisawa M, Humle T, McGrew WC, Nakamura M, Ohashi G, Yamanashi Y, Yamakoshi G, Matsuzawa T. 2015 Tools to tipple: ethanol ingestion by wild chimpanzees using leaf-sponges. R. Soc. Open Sci. **2**, 150150. (10.1098/rsos.150150) 26543588 PMC4632552

[9] Peris JE, Rodriguez A, Pena L, Fedriani JM. 2017 Fungal infestation boosts fruit aroma and fruit removal by mammals and birds. Sci. Rep. **7**, 5646. (10.1038/s41598-017-05643-z) 28717123 PMC5514155

[10] Janzen DH. 1977 Why fruits rot, seeds mold, and meat spoils. Am. Nat. **111**, 691-713. (10.1086/283200)

[11] Amato KR, Chaves OM, Mallot EK, Eppley TM, Abreu F, Barnett AA et al. 2021 Fermented food consumption in wild nonhuman primates and its ecological drivers. Am. J. Phys. Anthropol. **175**, 513-530. (10.1002/ajpa.24257) 33650680

[12] Di Fiore A, Link A, Campbell CJ. 2011 The Atelines: behavior and socioecological diversity in a New World monkey radiation. In Primates in perspective, 2nd edn (eds CJ Campbell, AF Fuentes, KC MacKinnon, R Stumpf, S Bearder), pp. 155-188. Oxford, UK: Oxford University Press.

[13] Campbell CJ. 2000 The reproductive biology of black-handed spider monkeys (*Ateles geoffroyi*): integrating behavior and endocrinology. Doctoral dissertation, University of California, Berkeley, CA.

[14] Ibañez DD, Salazar LT, Laska M. 2019 Taste responsiveness of spider monkeys to dietary ethanol, Chem. Senses **44**, 631-638. (10.1093/chemse/bjz049) 31400282

[15] Cant JGH. 1990 Feeding ecology of spider monkeys (*Ateles geoffroyi*) at Tikal, Guatemala. Hum. Evol. **5****,** 269-281. (10.1007/BF02437243)

[16] Felton AM, Felton A, Foley WJ, Lindenmayer DB. 2010 The role of timber tree species in the nutritional ecology of spider monkeys in a certified logging concession, Bolivia. Forest Ecol. Manage. **259****,** 1642-1649. (10.1016/j.foreco.2010.01.042)

[17] Benitez-Malvido J, Gonzalez-Di PM, Lombera R, Guillen S, Estrada A. 2014 Seed source, seed traits, and frugivore habits: implications for dispersal quality of two sympatric primates. Am. J. Bot. **101**, 970-978. (10.3732/ajb.1400147) 24920763

[18] Milton K. 2008 Macronutrient patterns of 19 species of Panamanian fruits from Barro Colorado Island. Neotrop. Primates **15**, 1-7. (10.1896/044.015.0101)

[19] Hiramatsu C, Melin AD, Aureli F, Schaffner CM, Vorobyev M, Kawamura S. 2009 Interplay of olfaction and vision in fruit foraging of spider monkeys. Anim. Behav. **77****,** 1421-1426. (10.1016/j.anbehav.2009.02.012)

[20] Nevo O, Garri RO, Hernandez Salazar LT, Schulz S, Heymann EW, Ayasse M, Laska M. 2015 Chemical recognition of fruit ripeness in spider monkeys (*Ateles geoffroyi*). Sci. Rep. **5**, 14895. (10.1038/srep14895) 26440380 PMC4594300

[21] Wurst FM, Wiesbeck GA, Metzger JW, Weinmann W, Graf M. 2004 On sensitivity, specificity, and the influence of various parameters on ethyl glucuronide levels in urine – results from the WHO/ISBRA Study. Alcohol. Clin. Exp. Res. **28****,** 1220-1228. (10.1097/01.ALC.0000134230.21414.11) 15318121

[22] Halter CC, Dresen S, Auwaerter V, Wurst, FM, Weinmann W. 2008 Kinetics in serum and urinary excretion of ethyl sulfate and ethyl glucuronide after medium dose ethanol intake. Int. J. Legal Med. **122**, 123-128. (10.1007/s00414-007-0180-8) 17558515

[23] Mercurio I, Politi P, Mezzetti E, Agostinelli F, Troiana G, Pellegrino A. 2021 Ethyl glucuronide and ethyl sulphate in urine: caution in their use as markers of recent alcohol use. Alcohol Alcoholism **56**, 201-209. (10.1093/alcalc/agaa113) 33170266

[24] Nevo O, Razafimandimby D, Jeffrey JAJ, Schulz S, Ayasse M. 2018 Fruit scent as an evolved signal to primate seed dispersal. Sci. Adv. **4**, aat4871. (10.1126/sciadv.aat4871) PMC6170039 30306132

[25] Yeomans MR. 2004. Effects of alcohol on food and energy intake in human subjects: evidence for passive and active over-consumption of energy. Br. J. Nutr. **92**, S31-S34. (10.1079/BJN20041139) 15384320

[26] Janiak MC, Pinto SL, Duytschaever G, Carrigan MA, Melin AD. 2020 Genetic evidence of widespread variation in ethanol metabolism among mammals: revisiting the ‘myth’ of natural intoxication. Biol. Lett. **16**, 20200070. (10.1098/rsbl.2020.0070) 32343936 PMC7211468

[27] Campbell CJ, Maro A, Weaver V, Dudley R. 2022 Dietary ethanol ingestion by free-ranging spider monkeys (*Ateles geoffroyi*). *Figshare*. 10.1098/rsos.211729 PMC8941420 35345427

